# Co-occurring obsessive–compulsive disorder and autism spectrum disorder in young people: prevalence, clinical characteristics and outcomes

**DOI:** 10.1007/s00787-020-01478-8

**Published:** 2020-02-01

**Authors:** Alex F. Martin, Amita Jassi, Alexis E. Cullen, Matthew Broadbent, Johnny Downs, Georgina Krebs

**Affiliations:** 1 Department of Psychology, Institute of Psychiatry, Psychology and Neuroscience, King’s College London, 16 De Crespigny Park, London, SE5 8AF UK; 2 OCD, BDD and Related Disorders Clinic, South London and Maudsley NHS Trust, London, UK; 3 Department of Psychosis Studies, Institute of Psychiatry, Psychology and Neuroscience, King’s College London, London, UK; 4 NIHR Biomedical Research Centre for Mental Health, South London and Maudsley NHS Foundation, London, UK; 5 Department of Child and Adolescent Psychiatry, Institute of Psychiatry, Psychology and Neuroscience, King’s College London, London, UK; 6 Social, Genetic and Developmental Psychiatry Centre, Institute of Psychiatry, Psychology and Neuroscience, King’s College London, London, UK

**Keywords:** Obsessive–compulsive disorder, Autism spectrum disorders, Comorbidity, Treatment outcomes, Psychosocial functioning

## Abstract

Obsessive–compulsive disorder (OCD) and autism spectrum disorders (ASD) commonly co-occur and are considered challenging to manage when they co-occur in youth. However, clinical characteristics and prognosis of this group remain poorly understood. This study examined the prevalence, clinical correlates and outcomes of paediatric OCD co-occurring with ASD (OCD + ASD) in a large clinical cohort. Data were extracted from electronic clinical records of young people aged 4–17 years who had attended a mental health trust in South London, United Kingdom. We identified young people with diagnoses of OCD + ASD (*n* = 335), OCD without ASD (*n* = 1010), and ASD without OCD (*n* = 6577). 25% of youth with OCD had a diagnosis of ASD, while 5% of those with ASD had a diagnosis of OCD. At diagnosis, youth with OCD + ASD had lower psychosocial functioning scores on the clinician-rated Child Global Assessment Scale (CGAS) compared to those with either OCD or ASD. Youth with OCD + ASD were equally likely to receive CBT compared to those with OCD but were more likely to be prescribed medication and use services for longer than either comparison group. Youth with OCD + ASD showed significant improvements in functioning (CGAS scores) after service utilisation but their gains were smaller than those with OCD. OCD + ASD commonly co-occur, conferring substantial impairment, although OCD may be underdiagnosed in youth with ASD. Young people with co-occurring OCD + ASD can make significant improvements in functioning with routine clinical care but are likely to remain more impaired than typically developing youth with OCD, indicating a need for longer-term support for these young people.

**Electronic supplementary material:**

The online version of this article (10.1007/s00787-020-01478-8) contains supplementary material, which is available to authorized users.

## Introduction

Obsessive–compulsive disorder (OCD) and autism spectrum disorder (ASD) are both debilitating conditions in young people but are widely viewed as being particularly challenging to manage when they co-occur. ASD in young people is associated with high rates of mental health conditions [1], with anxiety and phobic disorders estimated in 41% of young people with ASD [2]. It is estimated that 17–37% of young people with ASD also experience OCD symptoms [3, 4] although the proportion of youth with OCD who also meet diagnostic criteria for ASD is less clear. Several studies have suggested that young people with OCD and co-occurring ASD (hereafter referred to as OCD + ASD) have clinically distinct psychopathology and impairment compared to those with ASD or OCD only [5–7]. However, these study findings may lack generalisability as they are limited by small sample sizes and selective recruitment, for example, excluding youth with intellectual disabilities.

A robust evidence-base supports the efficacy of cognitive behaviour therapy (CBT) and selective serotonin reuptake inhibitors (SSRIs) in the treatment of paediatric OCD [8]. Further, there is early evidence that CBT for children and adolescents with ASD is efficacious in the reduction of anxiety symptoms including OCD [9, 10], as well as OCD specific symptoms [11, 12], although there is indication that these treatments may be underutilised in routine clinical care [13]. It remains unclear whether youth with OCD + ASD are being offered these recommended first-line treatments, and if so, whether their outcomes are comparable to typically developing youth with OCD. Previous research has shown that CBT is associated with a smaller reduction in OCD symptoms among those with co-occurring ASD compared to typically developing youth [11], but global functional outcomes of youth with OCD + ASD following routine clinical care is unknown. Combined, this study may provide insight into diagnostic and treatment practices for youth with OCD + ASD during routine clinical care, which has important implications for understanding detection, diagnosis and provision of support for this population. Further, this may provide crucial information for planning and delivering mental health services for youth with co-occurring OCD and ASD.

In summary, the lack of available information on prevalence in a clinical setting, clinical characteristics, treatment provision and functional outcomes of youth with OCD + ASD has made it difficult for service providers to plan and deliver adequate treatment resources to meet the needs of these young people and their families. The current study utilised clinical data from a large cohort of young people accessing child and adolescent mental health services (CAMHS) in the United Kingdom (UK) to address substantial gaps in the current literature. Specifically, we aimed to examine: (a) the rates of co-occurrence between OCD and ASD; (b) the clinical characteristics of youth with OCD + ASD; and (c) the treatment received by youth with OCD + ASD and their outcomes following service utilisation.

## Method

### Study setting

The study employed a retrospective open cohort design. The cohort comprised patients assessed within South London and Maudsley NHS Foundation Trust (SLaM) between 1st January 2007 and 31st December 2016. SLaM provides mental health services to young people resident in four South London boroughs (Croydon, Lambeth, Lewisham and Southwark). In addition, the national and specialist services for OCD and ASD accept referrals from across the UK. Broadly, CAMHS accept referrals for school age children (4–18 years; exceptional cases are accepted below this age) with neurodevelopmental disorders, and/or displaying emotional or behavioural difficulties. Youth are referred from primary care and child health, educational and social care services. All referrals are assessed within multidisciplinary teams and assigned diagnoses based on ICD-10 [14] criteria. A number of core assessments are completed for all patients including clinician ratings of global functional impairment. Diagnosis-specific measures are completed as indicated. SLaM CAMHS provides a range of treatments, including pharmacotherapy and psychological therapies.

Data were extracted from anonymised electronic patient records using the Clinical Record Interactive Search (CRIS) system [15, 16]. This system enables researchers to search anonymised records of over 250,000 service users, including over 35,000 child and adolescent patients [17], representing nearly all those who have been in contact with SLaM services since 2006.

### Sample

All cases who presented to SLaM services aged between 4 and 17 years were screened for ICD-10 diagnoses within clinician-recorded structured or free-text fields. Those with structured data recorded were included if they had at least one diagnosis of OCD (F42.0–F42.9) and/or ASD (F84.0–F84.9). Diagnostic status was primarily determined by extracting information from the diagnosis field in patient records. Missing structured diagnostic data was supplemented by a dedicated natural language processing (NLP) application deployed through GATE (Generalized Architecture for Text Engineering), which automatically codes ‘free-text’ diagnostic data from clinical text in correspondence and progress notes, as previously described [16, 18]. Briefly, the GATE application used a preselected list of diagnostic terms (‘Autism; Autistic; Autism Spectrum Disorder; Autistic Spectrum Disorder; Asperger’s; Pervasive Developmental Disorder; PDD’; ‘Obsessive Compulsive Disorder; Obsessive comps; OCD, Obsessive–Compulsive’), and used a rule-based algorithm to identify diagnostic terms within free-text fields (e.g. progress notes, correspondence, reports). The NLP application contextualises positive diagnoses including identifying whether the diagnosis was: a) confirmed by a clinician (coded as positive diagnosis); b) a potential differential diagnosis (coded as absent diagnosis); c) relating to a third person (coded as absent diagnosis). Details of a similar, validated diagnostic extraction method have been reported elsewhere [19].

The final sample comprised 7922 young people who were categorised into the three diagnostic groups as follows: OCD + ASD (*n* = 335), OCD (single diagnosis or any co-occurring diagnosis excluding ASD, *n* = 1010), or ASD (single diagnosis or any co-occurring diagnosis excluding OCD, *n* = 6577). ASD and OCD diagnoses were recorded at any point that the diagnosis appeared in the patient record, within the observational period. For each participant, the ‘point of diagnosis’ was defined as the first recorded OCD diagnosis for the OCD and OCD + ASD groups, and first recorded ASD diagnosis for the ASD group. The ‘follow-up point’ was defined as the end of the observation period, which could have been completion of treatment, turning 18 years old, or death.

### Measures

*Child Global Assessment Scale (CGAS)* The CGAS [20] is a clinician-rated measure of global functioning, clinicians completing CGAS assessments were from a range of professional backgrounds (e.g. psychiatrists, clinical psychologists and nurses). Ratings are assigned based on the patient’s level of emotional and behavioural functioning over the previous 3 months, excluding physical impairments. Scores range from 1 to 100, with 70 and above indicating normative functioning. CGAS scores were extracted at diagnosis and at the closest point to the end of the observation period (follow-up).

*Demographics* Age was calculated as the difference between date of birth and diagnosis date. Socioeconomic status was assessed using UK Census data which provided ‘small level’ (on average 400 households) deprivation scores [21], where a higher score equates to greater area deprivation. 14 ethnicity categories were collapsed into four to improve statistical sensitivity in line with previous research using CRIS [15].

*Revised Children’s Anxiety and Depression Scale (RCADS)* The RCADS [22] is a self-report measure of anxiety and depression. The RCADS has strong psychometric properties and has been validated for use with children with ASD [23, 24]. The OCD subscale includes three questions relating to obsessions (e.g. ‘I can’t seem to get bad or silly thoughts out of my head’) and three relating to compulsions (e.g. ‘I have to keep checking that I have done things right’). Items are rated on a 4-point Likert-type scale (‘never’ to ‘always’) with total scores ranging from 0 to 18. The OCD subscale has good psychometric properties in clinical populations, with high convergent validity against the OCD subscale of the Revised Children’s Manifest Anxiety Scale (*r* = 0.59, *p* < 0.01), good internal reliability (*α* = 0.82), and an acceptable test–retest coefficient (0.65) [25, 26]. OCD symptoms were measured at diagnosis.

The RCADS total score and subscales are reported as follows: separation anxiety; panic; depression; social anxiety; generalised anxiety. The subscales have good internal reliability (*α*s = 0.71–0.83) and good test–retest coefficients in clinical populations (0.75–0.80) [25, 26]. Total and subscale scores were measured at diagnosis.

*Intellectual disability status* Multiaxial ICD-10 Axis II diagnosis of Intellectual Disability (F70.x–F73.x) was extracted from structured data fields and collapsed into a binary variable (i.e. present or absent).

*Co-occurring psychiatric diagnosis* Multiaxial ICD-10 Axis II diagnoses were extracted from structured data fields and recorded as a binary variable (i.e. present or absent). These included any of the following disorders: affective (f3); phobic (f40); anxiety (f41); hyperkinetic (f90); conduct (f91); mixed conduct and emotion (f92); emotion (f93); tic (f95).

*National specialist CAMHS* A proportion was generated comparing young people in national specialist CAMHS compared to those in community CAMHS services.

*Treatment* Provision of CBT and pharmacotherapy [antidepressant; antipsychotic; sedative (hypnotic and anxiolytic e.g. benzodiazepine)] within 12 months of diagnosis was extracted from structured data fields, supplemented with free-text searches of correspondence and progress notes using NLP application.

*Time spent in services* We calculated the total number of days between diagnosis assessment and the end of the observation period (follow-up).

### Statistical analysis

All analyses were performed using Stata version 12.0 [27]. Two sets of group comparison analyses (*t* tests and chi-square tests) were conducted in which the OCD + ASD group was compared to: (a) the OCD group; and (b) the ASD group. All variables were assessed against test assumptions as appropriate. A mixed-model repeated measures ANCOVA was used to test the effects of diagnostic group (between-subjects factor) and timepoint (within-subjects factor: diagnosis versus follow-up). Covariates included age, sex, intellectual disability, area deprivation, treatment (pharmacotherapy and CBT), and time lapse between diagnosis and follow-up (days). ANCOVA results were explored using post-hoc Bonferroni corrected *t* tests.

## Results

### Rates of co-occurrence

Of the total sample (*n* = 7922), 335 youth (4.2%, 95% CI: 3.8–4.7) had a co-occurring diagnosis of OCD + ASD. Of the 6912 youth with a diagnosis of ASD, 335 (4.8% [95% CI: 4.4–5.4]) also had a diagnosis of OCD. Of the 1345 youth with a diagnosis of OCD, 335 (24.9% [95% CI: 22.6–27.3]) also had a diagnosis of ASD.

### Demographic and clinical characteristics at diagnosis

As shown in Table 1, the mean age of OCD diagnosis was significantly younger in the OCD + ASD group (13 years 6 months) than in the OCD group (14 years). The age of ASD diagnosis was significantly older in the OCD + ASD group (13 years 4 months) compared to the ASD group (10 years 9 months). The greatest prevalence of intellectual disability was observed in the ASD group (20%), followed by OCD + ASD group (9%), whilst the OCD group had the lowest prevalence (1%). The prevalence of any other co-occurring psychiatric diagnosis for the OCD + ASD group was 23%. There was no significant difference in the prevalence compared to the OCD group. However, the odds of youth in the OCD + ASD group having at least one other co-occurring diagnosis was 2.06 times greater than those in the ASD group. The OCD + ASD group had the highest proportion of White British children (73%), followed by the OCD group (70%) and then the ASD group (54%). Black children were overrepresented in the ASD group compared to expected frequencies in the sampled population and underrepresented in the OCD + ASD and OCD groups. At diagnosis, young people in the OCD + ASD group presented with significantly greater RCADS OCD symptom scores (*M* = 7.48) compared to those with ASD (*M* = 5.72), but significantly lower OCD symptom scores compared to those with OCD (*M* = 9.30). No differences were identified between the groups for any other RCADS subscales scores or total score. At diagnosis, youth with OCD + ASD presented with greater functional impairment as indicated by significantly lower CGAS scores (*M* = 44.30) compared to those with OCD (*M* = 49.06) and ASD (*M* = 48.87).

**Table 1:** Demographic and clinical characteristics of youth with OCD + ASD, OCD and ASD at diagnosis

	OCD + ASD			OCD					ASD				
	*N* = 335			*N* = 1010					*N* = 6577				
						OCD + ASD compared to OCD group					OCD + ASD compared to ASD group		
	Mean	SD		Mean	SD	*t*	*p*	*d*	Mean	SD	*t*	*p*	*d*
Age at ASD diagnosis^a^	13.28	3.01		–	–	–	–	–	10.76	3.72	12.16	***	0.74
Age at OCD diagnosis^a^	13.47	2.81		14.00	2.59	− 3.19	**	0.20	–	–	–	–	–
Area deprivation	20.88	12.75		22.36	12.44	− 1.85	ns	0.18	27.11	12.15	− 9.02	***	0.50
CGAS score	44.30	3.29		49.06	15.57	− 5.35	***	0.42	48.87	14.60	− 5.66	***	0.45
RCADS scores													
Total	49.44	31.23		56.68	30.90	− 1.32	ns	0.23	49.68	28.44	− 0.05	ns	0.01
OCD	7.48	5.29		9.30	5.26	− 2.05	*	0.35	5.72	4.19	2.68	**	0.37
Separation anxiety	11.11	8.25		11.81	7.32	− 0.53	ns	0.01	12.04	7.16	− 0.82	ns	0.12
Panic	8.27	7.50		8.42	6.63	− 0.13	ns	0.02	7.28	6.45	0.97	ns	0.14
Depression	12.28	6.56		11.42	7.50	0.71	ns	0.12	11.86	7.04	0.39	ns	0.06
Social anxiety	5.34	4.71		6.49	5.10	− 1.34	ns	0.23	5.93	4.77	− 0.78	ns	0.12
Generalised anxiety	7.47	5.29		8.89	5.46	− 1.54	ns	0.26	7.34	4.79	0.17	ns	0.03

						OCD + ASD compared to OCD group					OCD + ASD compared to ASD group		
	*N*	Percent		*N*	Percent	chi^2^	*p*	OR	*N*	Percent	chi^2^	*p*	OR
Intellectual disability	29	9		11	1	49.93	***	8.61	1302	20	25.44	***	0.38
Sex (male)	214	64		488	49	24.42	***	1.89	4952	76	21.99	***	0.58
Other psychiatric diagnosis	77	23		247	25	0.30	ns	0.92	834	13	29.58	***	2.06

Ethnicity	*N*	Percent	Expected *N*^b^	*N*	Percent	Expected *N*^b^			*N*	Percent	Expected *N*^b^		
White British	234	73	180.5	656	70	532.5			3070	54	3247		
Black	13	3	68.4	71	8	201.8			1417	25	1230.7		
Asian	20	6	42.8	46	5	42.8			252	4	260.7		
Other	52	16	163.9	168	18	163.9			999	17	999.5		

CGAS score adjusted for age, sex, intellectual disability, area deprivation, treatment (pharmacotherapy and cognitive behavioural therapy), time between assessments

*OCD* obsessive–compulsive disorder, *ASD* autism spectrum disorder, *CGAS* Child Global Assessment Scale, *RCADS* Revised Children’s Anxiety and Depression Scale, *ns* non-significant

***p < 0.001; **p < 0.01; *p < 0.05

^a^First recorded diagnosis

^b^Expected *N* based on ethnic composition of local population obtained from UK Census data

### Treatment received and clinical outcomes

As shown in Table 2, the OCD + ASD group were engaged with services for a mean of 632 days, which was significantly longer than the OCD (441 days) or ASD group (484 days). 68% of young people in the OCD + ASD group were in national specialist CAMHS, compared to 49% in the OCD group and 28% from the ASD group. A higher proportion of the OCD + ASD group were prescribed antidepressant, antipsychotic and sedative medications compared to the OCD and ASD groups. The odds of young people with OCD + ASD being prescribed antidepressants was 11 times greater than those with ASD (OR = 11.28) and 2.5 times greater than those with OCD (OR = 2.57). The odds of young people with OCD + ASD being prescribed antipsychotics was almost 3 times greater than those with ASD (OR = 2.74) and those with OCD (OR = 2.87), and for sedatives, the odds were 1.5 times greater for young people with OCD + ASD than those with ASD (OR = 1.43) and three times greater than those with OCD (OR = 2.99). The OCD + ASD group were equally likely to receive CBT compared to the OCD group, but more likely than the ASD group.

**Table 2:** Treatment received and outcomes for youth with OCD + ASD, OCD and ASD

	OCD + ASD		OCD					ASD				
	*N* = 335		*N* = 1010					*N* = 6577				
					OCD + ASD compared to OCD group					OCD + ASD compared to ASD group		
	Mean	SD	Mean	SD	*t*	*p*	*d*	Mean	SD	*t*	*p*	*d*
Time in services (days)	631.71	597.68	441.42	432.61	5.77	***	0.37	484.37	648.75	3.76	***	0.24
CGAS at follow-up	54.20	15.01	61.36	16.21	− 7.68	***	0.46	53.54	16.22	0.77	*	0.04
CGAS change score	8.84	18.30	12.03	16.84	− 3.49	***	0.18	5.62	17.03	3.79	***	0.18

					OCD + ASD compared to OCD group					OCD + ASD compared to ASD group		
	*N*	Percent	*N*	Percent	chi^2^	*p*	OR	*N*	Percent	chi^2^	*p*	OR
National Specialist CAMHS	199	59	500	49	9.87	**	1.49	1860	28	147.63	***	3.71
Antidepressant	227	68	454	45	52.37	***	2.57	1033	16	579.47	***	11.28
Antipsychotic	117	35	159	16	56.76	***	2.87	1076	16	76.93	***	2.74
Sedative	91	27	112	11	50.73	***	2.99	1363	21	7.96	**	1.43
CBT	161	48	531	53	2.05	ns	0.83	484	8	624.11	***	11.65

CGAS score adjusted for age, sex, intellectual disability, area deprivation, treatment (pharmacotherapy and cognitive behavioural therapy), time between assessments

*OCD* obsessive–compulsive disorder, *ASD* autism spectrum disorder, *CGAS* Child Global Assessment Scale, *ns* non-significant

***p < 0.001; **p < 0.01; *p < 0.05

**Fig. 1:**
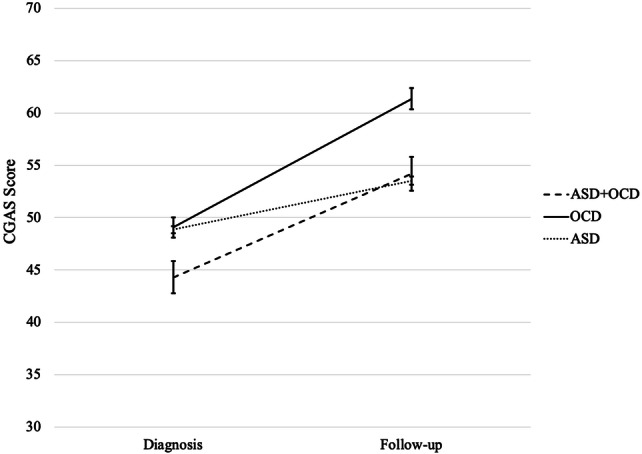
Means and 95% confidence intervals for functional deficit score by group at diagnosis and follow-up, controlling for age, sex, intellectual disability, area deprivation, treatment (pharmacotherapy and cognitive behavioural therapy), time between assessments. Scores range from 1 to 100: > 70 indicates normative functioning

Functional impairment scores at follow-up and their change from diagnosis to follow-up are shown in Table 2 and Fig. 1. A mixed-model ANCOVA (Table 3) was conducted to determine the effect of timepoint (diagnosis versus follow-up), diagnostic group (OCD + ASD versus OCD versus ASD), and their interaction on functional impairment. Analyses controlled for age, sex, intellectual disability, area deprivation, treatment (pharmacotherapy and CBT), and time lapse between diagnosis and follow-up (days). The ANCOVA revealed a main effect of group (*F*(2,14) = 255.44, *p* < 0.001), indicating that functional impairment scores were significantly different between the groups. There was also a main effect of timepoint (*F*(1,14) = 422.68, *p* < 0.001), showing a significant improvement in psychosocial functioning after utilising CAMHS care. Importantly, there was also a significant group × timepoint interaction (*F*(2,14) = 65.36, *p* < 0.001), showing that the extent to which functional impairment improved differed between the groups. Post hoc *t* tests were used to decompose these interaction effects (see Table 2). The OCD + ASD group showed a significantly smaller improvement in functioning (*M* = 8.84) compared to the OCD group (*M* = 12.03), but a greater improvement than the ASD group (*M* = 5.62). At follow-up, youth with OCD + ASD had greater functional impairment (*M* = 54.20) than those with OCD (*M* = 61.36), but a comparable level to those with ASD (*M* = 53.54).

**Table 3:** Mixed ANCOVA modelling effects of diagnostic group (OCD + ASD, OCD versus ASD) and time (diagnosis versus follow-up) on functional impairments (CGAS score)

	*df*	*F*	*p*
Independent variables			
Group	2	61.38	< 0.001
Time	1	422.68	< 0.001
Group × time	2	65.36	< 0.001
Covariates			
Age	1	20.09	< 0.001
Sex	1	0.00	0.957
Area deprivation	1	0.25	0.617
Intellectual disability	1	1224.65	< 0.001
Antidepressant	1	118.35	< 0.001
Antipsychotic	1	315.60	< 0.001
Sedative	1	24.62	< 0.001
CBT	1	38.60	< 0.001
Time difference	1	55.70	< 0.001

*OCD* obsessive–compulsive disorder, *ASD* autism spectrum disorder

## Discussion

In the largest study of co-occurring OCD and ASD to date, we found that approximately 25% of young people with OCD also had a diagnosis of ASD. To our knowledge, this is the first prevalence estimate of ASD in a clinical population of youth with OCD. Of note, the high rate of co-occurrence observed in this study may partly reflect the inclusion of specialist ASD and OCD services which assess and treat more complex cases, including those with co-occurring psychiatric diagnoses. Of those young people who had a diagnosis of ASD, approximately 5% also had an OCD diagnosis. This figure was considerably lower than expected, although previous estimates were based on studies that systematically screened for OCD in ASD populations [3, 4]. Thus, it is possible that OCD is underdiagnosed in youth with ASD in routine clinical practice, because either ASD associated problems overshadow OCD symptoms, or OCD symptoms are viewed as part of ASD themselves.

In relation to our second aim, we found that several demographic and clinical features differentiated youth with OCD + ASD from those with OCD. Specifically, those with OCD + ASD tended to be younger at the point of OCD diagnosis, were more likely to be male, and were more likely to have an intellectual disability. Youth with OCD + ASD were equally likely to have an additional psychiatric diagnosis compared to those with OCD, but were more likely to have a co-occurring diagnosis than those with ASD alone. In the current study, we found lower OCD symptom severity among youth with OCD + ASD compared to those with OCD. This may reflect the fact that OCD symptoms were assessed using a self-report measure, and some young people with ASD may lack insight into their symptoms and/or may be less accurate at self-reporting [28], thus artificially lowering their OCD symptom score. We found that functional impairment was significantly greater in youth with OCD + ASD relative to those with OCD, consistent with previous findings [5]. Importantly, we also found that those with OCD + ASD were more impaired than youth with ASD. This difference did not appear to be accounted for by other symptoms such as anxiety, or the presence of co-occurring diagnoses, since these were comparable between the groups. Thus, our findings suggest that OCD and ASD are both impairing in their own right, but there is a cumulative burden of suffering from OCD and ASD together.

With respect to our third aim, we found that youth with OCD + ASD were engaged in mental health services for significantly longer than those with OCD or ASD only. Encouragingly, youth with OCD + ASD were equally likely to receive CBT compared to those with OCD only. However, it is notable that only half of the children in both groups accessed CBT; given that CBT is the recommended first-line treatment for paediatric OCD [8]. We found high rates of pharmacotherapy use in the OCD + ASD group, with 68% being prescribed antidepressant, 35% antipsychotic, and 27% sedative medication. Young people with OCD + ASD were considerably more likely to be prescribed these medications compared to those with OCD or ASD. This discrepancy is unlikely to be accounted for by OCD severity, especially given that youth with OCD + ASD scored lower on the measure of OCD symptoms at diagnosis compared to those with OCD. It is also unlikely that the presence of co-occurring diagnoses drives higher rates of medications, as there was no difference in diagnosis rates between the OCD + ASD and OCD groups. However, it is possible that OCD symptoms have a different impact on youth with ASD compared to those without, such as greater agitation or insomnia, as well as a greater functional impairment, leading to increased use of medication.

In this study, we found that psychosocial functioning improved significantly for youth with OCD + ASD following utilisation of mental health services, but that their functional gains were smaller than youth with OCD only. This is in line with previous findings that that OCD is a treatable condition but that youth with co-occurring ASD often have poorer outcomes than their typically developing counterparts [11]. We additionally observed that by follow-up, youth with OCD + ASD were no longer more impaired than those with ASD only, tentatively suggesting that OCD symptoms were successfully treated in the OCD + ASD group, while ASD symptomatology remained. However, we are unable to directly test this hypothesis as OCD symptom measures were not available at follow-up.

The current study has several notable strengths. It is the first study to utilise a large, representative clinical cohort to simultaneously examine youth with OCD + ASD, OCD and ASD. Both of the comparison groups (OCD or ASD) were allowed to contain co-occurring diagnoses (excluding the other diagnosis of interest), meaning that our findings are more generalisable to typical clinical settings. Similarly, this study did not exclude participants on the basis of intellectual disability, making the results generalisable to the wider ASD population. Nevertheless, our results should also be considered in the context of some limitations. We did not have detailed information regarding medication dose or duration, or CBT focus or duration. Furthermore, as treatment information was limited to CBT and prescribed medications, it is possible that young people with ASD were offered a range of psychosocial interventions which were not captured in this study. Given that the primary outcome measure was a clinician-rated global functioning scale, it would be desirable to replicate this study using multiple-informant measures across a range of functional and symptom domains. Lastly, further research is needed to test whether the current findings generalise to other countries and healthcare systems.

## Implications

Our results have a number of important clinical implications. Our finding that approximately a quarter of young people with OCD also met diagnostic criteria for ASD highlights the need for clinicians working with paediatric OCD to be have adequate knowledge of ASD. This is important to ensure accurate detection, diagnosis and provision of support for this population. In the current study, we found that a smaller proportion of youth with ASD (5%) had a concurrent diagnosis of OCD than expected based on previous studies in community samples of youth with ASD [3]. This finding could suggest that OCD is underdiagnosed in young people with ASD in routine clinical practice, possibly because either ASD associated problems overshadow OCD symptoms [2], or OCD symptoms are viewed as part of ASD themselves [29]. This emphasizes the importance of clinicians being vigilant to OCD symptoms in young people with ASD.

Psychosocial functioning was most impaired at diagnosis for young people with OCD + ASD compared to those with either OCD or ASD, suggesting a cumulative burden of suffering OCD concurrent with ASD. However, functioning not only improved after accessing mental health services, but by follow-up, young people with OCD + ASD were no longer more functionally impaired than those with ASD. These findings highlight the value of youth with OCD + ASD accessing mental health services, and therefore the importance of appropriate referral. However, we identified that only half of young people with OCD (as a primary diagnosis or concurrent with ASD) accessed CBT. As CBT is indicated for first-line treatment for paediatric OCD, this finding highlights the need for increased CBT dissemination efforts within clinical practice.

## Electronic supplementary material

Below is the link to the electronic supplementary material.

Supplementary file1 (DOCX 15 kb)
