# Supplementary material for: Co-occurring obsessive–compulsive disorder and autism spectrum disorder in young people: prevalence, clinical characteristics and outcomes
Source: Eur Child Adolesc Psychiatry. 2020 Feb 1;29(11):1603–11. doi: 10.1007/s00787-020-01478-8 (PMC7595977; doi:10.1007/s00787-020-01478-8)
Supplement: Supplementary file 1 — Supplementary file1 (DOCX 15 kb) [file 787_2020_1478_MOESM1_ESM.docx]

**Supplementary materials**

**Supplementary Table S1** RCADS item scores for youth with OCD+ASD, OCD and ASD at diagnosis

|  |  | OCD+ASD | |  | OCD | | | |  | ASD | | | |
| --- | --- | --- | --- | --- | --- | --- | --- | --- | --- | --- | --- | --- | --- |
|  |  | *N* = 335 | |  | *N* = 1010 | | | |  | *N* = 6577 | | | |
|  |  |  |  |  |  |  | OCD+ASD compared to OCD group | |  |  |  | OCD+ASD compared to ASD group | |
|  |  |  |  |  |  |  |  |  |  |  |  |  |  |
|  |  | Mean | *SD* |  | Mean | *SD* | *t* | *p* |  | Mean | *SD* | *t* | *p* |
| Obsessions | |  |  |  |  |  |  |  |  |  |  |  |  |
|  | Q10 Bothered by thoughts | 1.52 | 1.15 |  | 1.79 | 1.11 | -1.37 | *ns* |  | 1.20 | 1.09 | 1.90 | *ns* |
|  | Q23 Can't get thoughts out of head | 1.41 | 1.04 |  | 1.81 | 1.10 | -2.14 | * |  | 1.24 | 1.06 | 0.99 | *ns* |
| Compulsions | |  |  |  |  |  |  |  |  |  |  |  |  |
|  | Q31 Thoughts to stop bad things | 0.77 | 0.90 |  | 1.09 | 1.18 | -1.65 | *ns* |  | 0.59 | 0.89 | 1.28 | *ns* |
|  | Q16 Checking | 1.66 | 1.18 |  | 1.66 | 1.13 | -0.00 | *ns* |  | 1.19 | 1.05 | 2.82 | ** |
|  | Q42 Repeat behaviours | 1.48 | 1.05 |  | 1.68 | 1.13 | -1.07 | *ns* |  | 0.96 | 0.10 | 3.33 | ***** |
|  | Q44 Behaviours to stop bad things | 1.19 | 1.12 |  | 1.56 | 1.21 | -1.81 | *ns* |  | 0.84 | 0.95 | 2.25 | *** |

RCADS = Revised Children’s Anxiety and Depression Scale; OCD = obsessive-compulsive disorder; ASD = autism spectrum disorder. *** p < .001; ** p < .01; * p < .05; ns = non
